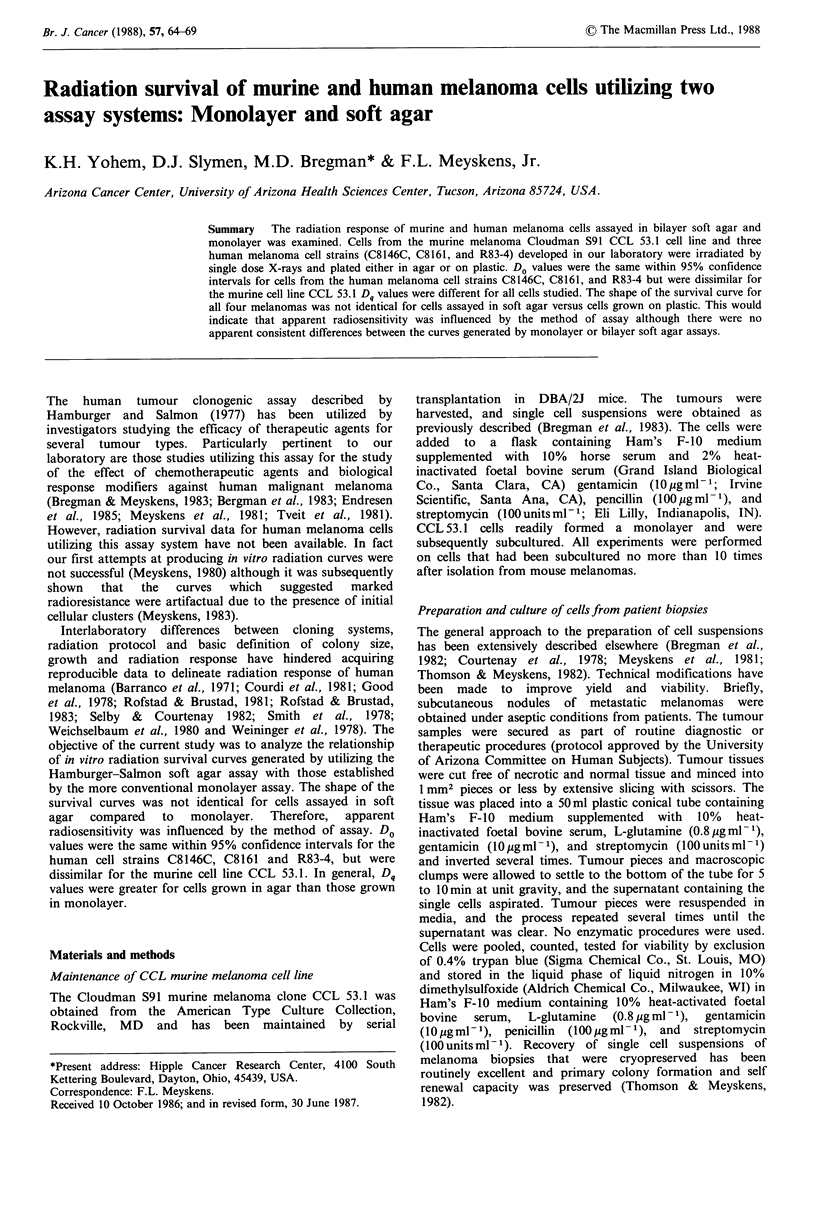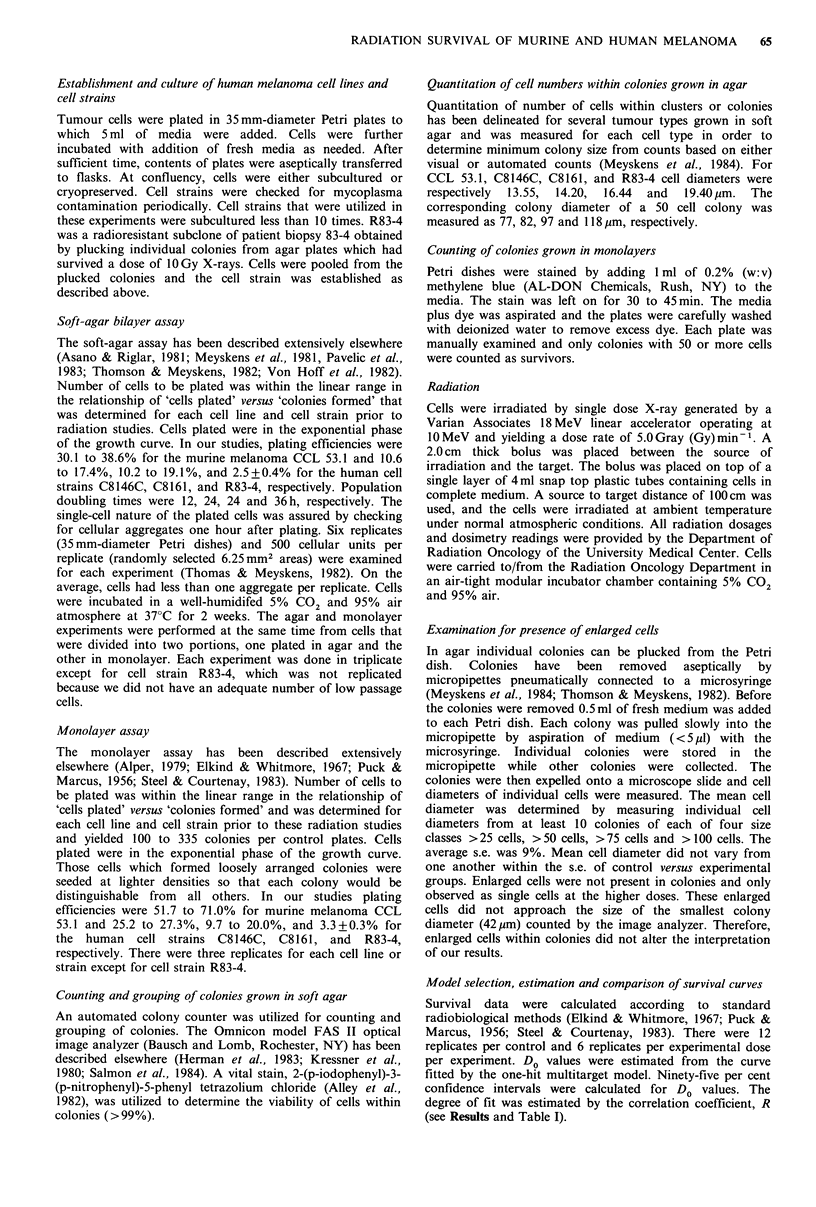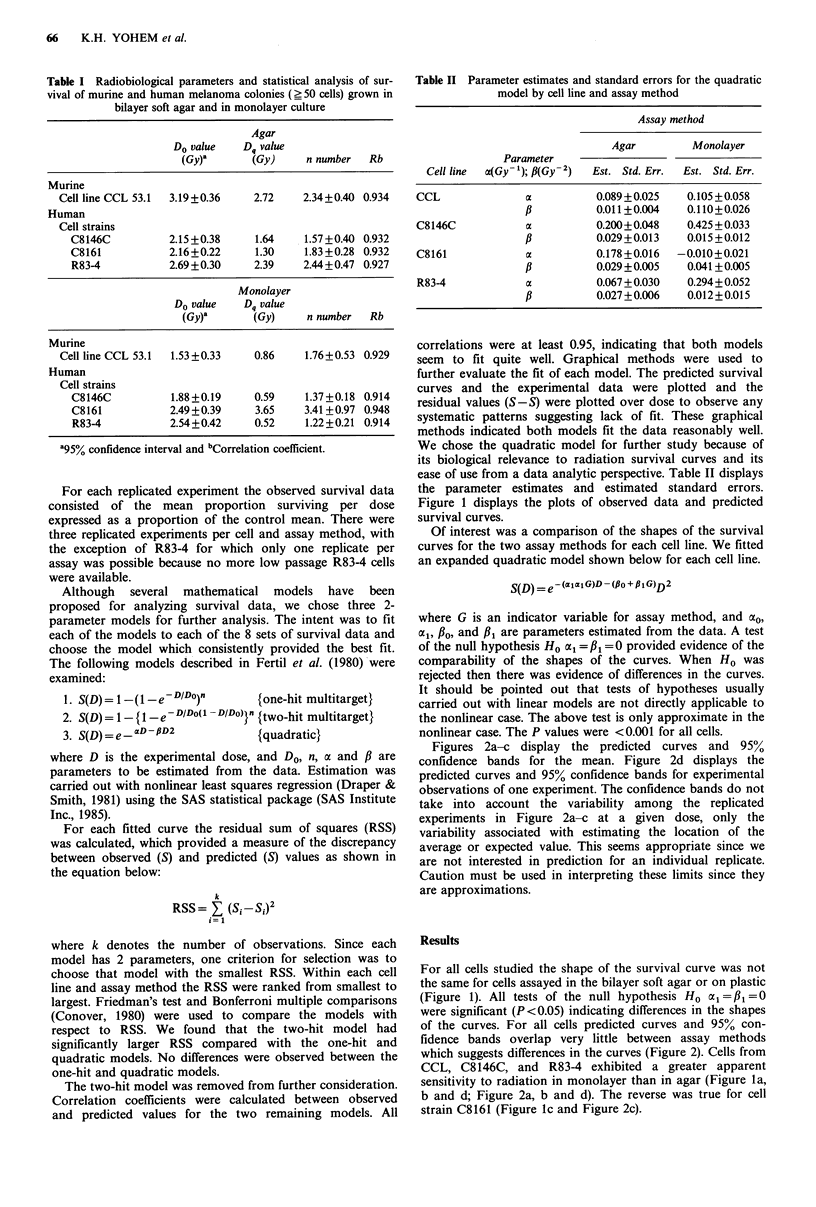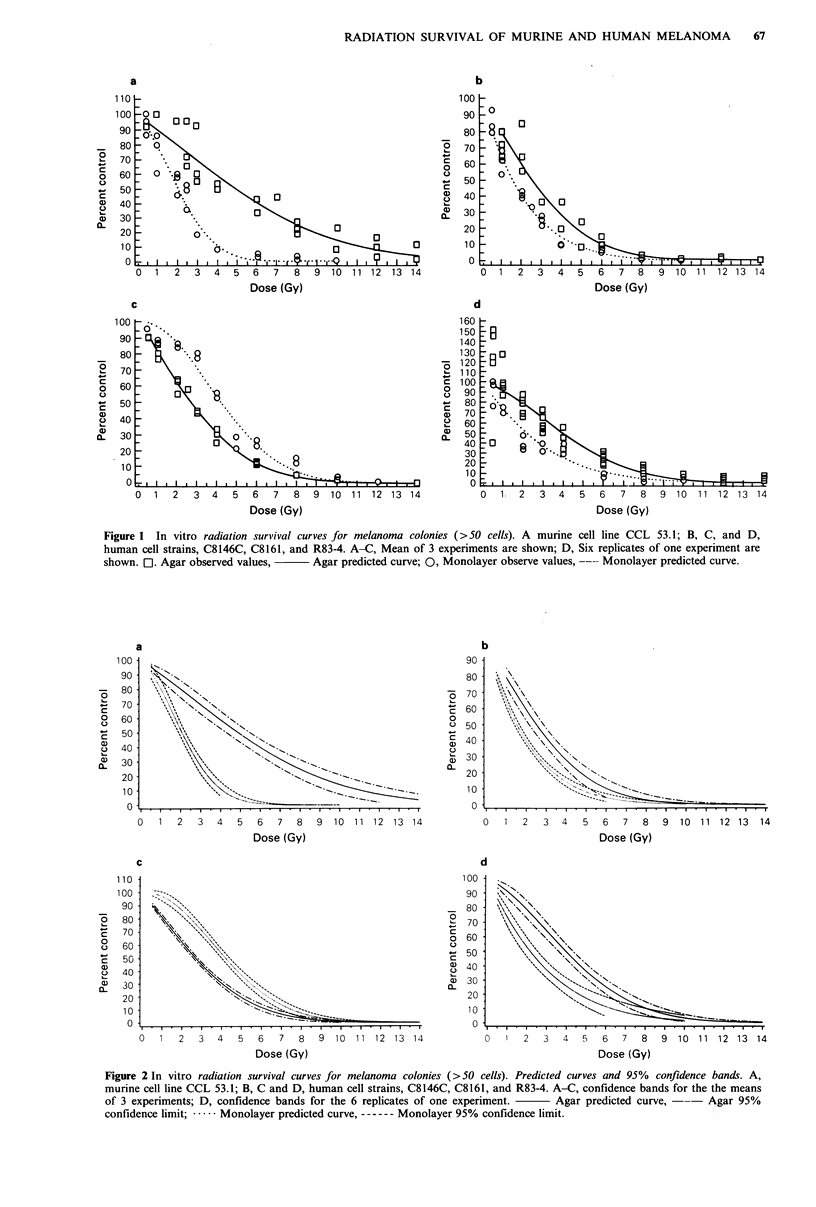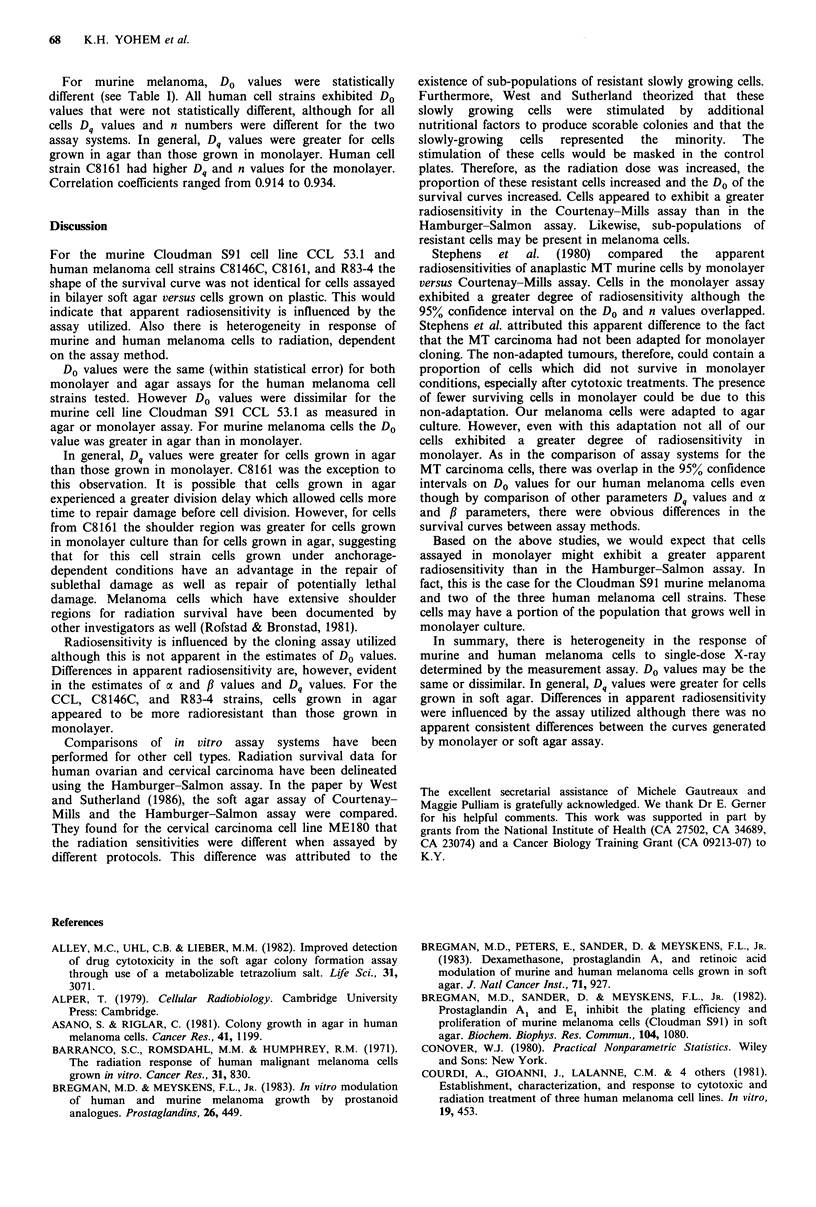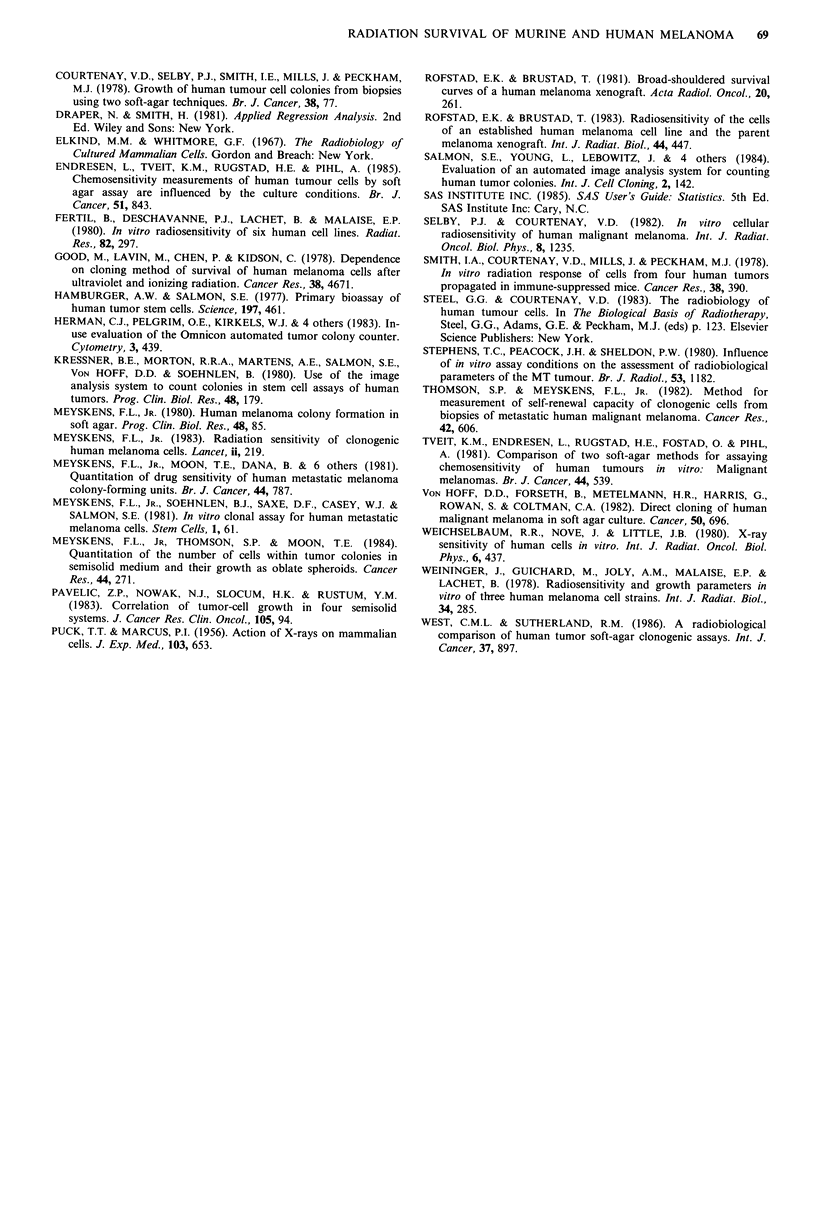# Radiation survival of murine and human melanoma cells utilizing two assay systems: monolayer and soft agar.

**DOI:** 10.1038/bjc.1988.10

**Published:** 1988-01

**Authors:** K. H. Yohem, D. J. Slymen, M. D. Bregman, F. L. Meyskens

**Affiliations:** Arizona Cancer Center, University of Arizona Health Sciences Center, Tucson 85724.

## Abstract

The radiation response of murine and human melanoma cells assayed in bilayer soft agar and monolayer was examined. Cells from the murine melanoma Cloudman S91 CCL 53.1 cell line and three human melanoma cell strains (C8146C, C8161, and R83-4) developed in our laboratory were irradiated by single dose X-rays and plated either in agar or on plastic. D0 values were the same within 95% confidence intervals for cells from the human melanoma cell strains C8146C, C8161, and R83-4 but were dissimilar for the murine cell line CCL 53.1 Dq values were different for all cells studied. The shape of the survival curve for all four melanomas was not identical for cells assayed in soft agar versus cells grown on plastic. This would indicate that apparent radiosensitivity was influenced by the method of assay although there were no apparent consistent differences between the curves generated by monolayer or bilayer soft agar assays.


Br. J. Cancer (1988), 57, 64–69 © The Macmillan Press Ltd., 1988

### Radiation survival of murine and human melanoma cells utilizing two assay systems: Monolayer and soft agar

K.H. Yohem, D.J. Slymen, M.D. Bregman* & F.L. Meyskens, Jr.

*Arizona Cancer Center, University of Arizona Health Sciences Center, Tucson, Arizona 85724, USA.*

**Summary** The radiation response of murine and human melanoma cells assayed in bilayer soft agar and monolayer was examined. Cells from the murine melanoma Cloudman S91 CCL 53.1 cell line and three human melanoma cell strains (C8146C, C8161, and R83-4) developed in our laboratory were irradiated by single dose X-rays and plated either in agar or on plastic. $D_0$ values were the same within 95% confidence intervals for cells from the human melanoma cell strains C8146C, C8161, and R83-4 but were dissimilar for the murine cell line CCL 53.1 $D_q$ values were different for all cells studied. The shape of the survival curve for all four melanomas was not identical for cells assayed in soft agar versus cells grown on plastic. This would indicate that apparent radiosensitivity was influenced by the method of assay although there were no apparent consistent differences between the curves generated by monolayer or bilayer soft agar assays.

The human tumour clonogenic assay described by Hamburger and Salmon (1977) has been utilized by investigators studying the efficacy of therapeutic agents for several tumour types. Particularly pertinent to our laboratory are those studies utilizing this assay for the study of the effect of chemotherapeutic agents and biological response modifiers against human malignant melanoma (Bregman & Meyskens, 1983; Bergman *et al.*, 1983; Endresen *et al.*, 1985; Meyskens *et al.*, 1981; Tveit *et al.*, 1981). However, radiation survival data for human melanoma cells utilizing this assay system have not been available. In fact our first attempts at producing *in vitro* radiation curves were not successful (Meyskens, 1980) although it was subsequently shown that the curves which suggested marked radioresistance were artifactual due to the presence of initial cellular clusters (Meyskens, 1983).

Interlaboratory differences between cloning systems, radiation protocol and basic definition of colony size, growth and radiation response have hindered acquiring reproducible data to delineate radiation response of human melanoma (Barranco *et al.*, 1971; Courdi *et al.*, 1981; Good *et al.*, 1978; Rofstad & Brustad, 1981; Rofstad & Brustad, 1983; Selby & Courtenay 1982; Smith *et al.*, 1978; Weichselbaum *et al.*, 1980 and Weininger *et al.*, 1978). The objective of the current study was to analyze the relationship of *in vitro* radiation survival curves generated by utilizing the Hamburger–Salmon soft agar assay with those established by the more conventional monolayer assay. The shape of the survival curves was not identical for cells assayed in soft agar compared to monolayer. Therefore, apparent radiosensitivity was influenced by the method of assay. $D_0$ values were the same within 95% confidence intervals for the human cell strains C8146C, C8161 and R83-4, but were dissimilar for the murine cell line CCL 53.1. In general, $D_q$ values were greater for cells grown in agar than those grown in monolayer.

### Materials and methods

### *Maintenance of CCL murine melanoma cell line*

The Cloudman S91 murine melanoma clone CCL 53.1 was obtained from the American Type Culture Collection, Rockville, MD and has been maintained by serial transplantation in DBA/2J mice. The tumours were harvested, and single cell suspensions were obtained as previously described (Bregman *et al.*, 1983). The cells were added to a flask containing Ham's F-10 medium supplemented with 10% horse serum and 2% heat-inactivated foetal bovine serum (Grand Island Biological Co., Santa Clara, CA) gentamicin (10 $\mu$g ml$^{-1}$; Irvine Scientific, Santa Ana, CA), pencillin (100 $\mu$g ml$^{-1}$), and streptomycin (100 units ml$^{-1}$; Eli Lilly, Indianapolis, IN). CCL 53.1 cells readily formed a monolayer and were subsequently subcultured. All experiments were performed on cells that had been subcultured no more than 10 times after isolation from mouse melanomas.

### *Preparation and culture of cells from patient biopsies*

The general approach to the preparation of cell suspensions has been extensively described elsewhere (Bregman *et al.*, 1982; Courtenay *et al.*, 1978; Meyskens *et al.*, 1981; Thomson & Meyskens, 1982). Technical modifications have been made to improve yield and viability. Briefly, subcutaneous nodules of metastatic melanomas were obtained under aseptic conditions from patients. The tumour samples were secured as part of routine diagnostic or therapeutic procedures (protocol approved by the University of Arizona Committee on Human Subjects). Tumour tissues were cut free of necrotic and normal tissue and minced into 1 mm$^2$ pieces or less by extensive slicing with scissors. The tissue was placed into a 50 ml plastic conical tube containing Ham's F-10 medium supplemented with 10% heat-inactivated foetal bovine serum, L-glutamine (0.8 $\mu$g ml$^{-1}$), gentamicin (10 $\mu$g ml$^{-1}$), and streptomycin (100 units ml$^{-1}$) and inverted several times. Tumour pieces and macroscopic clumps were allowed to settle to the bottom of the tube for 5 to 10 min at unit gravity, and the supernatant containing the single cells aspirated. Tumour pieces were resuspended in media, and the process repeated several times until the supernatant was clear. No enzymatic procedures were used. Cells were pooled, counted, tested for viability by exclusion of 0.4% trypan blue (Sigma Chemical Co., St. Louis, MO) and stored in the liquid phase of liquid nitrogen in 10% dimethylsulfoxide (Aldrich Chemical Co., Milwaukee, WI) in Ham's F-10 medium containing 10% heat-activated foetal bovine serum, L-glutamine (0.8 $\mu$g ml$^{-1}$), gentamicin (10 $\mu$g ml$^{-1}$), penicillin (100 $\mu$g ml$^{-1}$), and streptomycin (100 units ml$^{-1}$). Recovery of single cell suspensions of melanoma biopsies that were cryopreserved has been routinely excellent and primary colony formation and self renewal capacity was preserved (Thomson & Meyskens, 1982).

*Present address: Hipple Cancer Research Center, 4100 South Kettering Boulevard, Dayton, Ohio, 45439, USA.
Correspondence: F.L. Meyskens.
Received 10 October 1986; and in revised form, 30 June 1987.

*Establishment and culture of human melanoma cell lines and cell strains*

Tumour cells were plated in 35 mm-diameter Petri plates to which 5 ml of media were added. Cells were further incubated with addition of fresh media as needed. After sufficient time, contents of plates were aseptically transferred to flasks. At confluency, cells were either subcultured or cryopreserved. Cell strains were checked for mycoplasma contamination periodically. Cell strains that were utilized in these experiments were subcultured less than 10 times. R83-4 was a radioresistant subclone of patient biopsy 83-4 obtained by plucking individual colonies from agar plates which had survived a dose of 10 Gy X-rays. Cells were pooled from the plucked colonies and the cell strain was established as described above.

*Soft-agar bilayer assay*

The soft-agar assay has been described extensively elsewhere (Asano & Riglar, 1981; Meyskens *et al.*, 1981, Pavelic *et al.*, 1983; Thomson & Meyskens, 1982; Von Hoff *et al.*, 1982). Number of cells to be plated was within the linear range in the relationship of 'cells plated' *versus* 'colonies formed' that was determined for each cell line and cell strain prior to radiation studies. Cells plated were in the exponential phase of the growth curve. In our studies, plating efficiencies were 30.1 to 38.6% for the murine melanoma CCL 53.1 and 10.6 to 17.4%, 10.2 to 19.1%, and 2.5±0.4% for the human cell strains C8146C, C8161, and R83-4, respectively. Population doubling times were 12, 24, 24 and 36 h, respectively. The single-cell nature of the plated cells was assured by checking for cellular aggregates one hour after plating. Six replicates (35 mm-diameter Petri dishes) and 500 cellular units per replicate (randomly selected 6.25 mm$^2$ areas) were examined for each experiment (Thomas & Meyskens, 1982). On the average, cells had less than one aggregate per replicate. Cells were incubated in a well-humidifed 5% $CO_2$ and 95% air atmosphere at 37°C for 2 weeks. The agar and monolayer experiments were performed at the same time from cells that were divided into two portions, one plated in agar and the other in monolayer. Each experiment was done in triplicate except for cell strain R83-4, which was not replicated because we did not have an adequate number of low passage cells.

*Monolayer assay*

The monolayer assay has been described extensively elsewhere (Alper, 1979; Elkind & Whitmore, 1967; Puck & Marcus, 1956; Steel & Courtenay, 1983). Number of cells to be plated was within the linear range in the relationship of 'cells plated' *versus* 'colonies formed' and was determined for each cell line and cell strain prior to these radiation studies and yielded 100 to 335 colonies per control plates. Cells plated were in the exponential phase of the growth curve. Those cells which formed loosely arranged colonies were seeded at lighter densities so that each colony would be distinguishable from all others. In our studies plating efficiencies were 51.7 to 71.0% for murine melanoma CCL 53.1 and 25.2 to 27.3%, 9.7 to 20.0%, and 3.3±0.3% for the human cell strains C8146C, C8161, and R83-4, respectively. There were three replicates for each cell line or strain except for cell strain R83-4.

*Counting and grouping of colonies grown in soft agar*

An automated colony counter was utilized for counting and grouping of colonies. The Omnicon model FAS II optical image analyzer (Bausch and Lomb, Rochester, NY) has been described elsewhere (Herman *et al.*, 1983; Kressner *et al.*, 1980; Salmon *et al.*, 1984). A vital stain, 2-(p-iodophenyl)-3-(p-nitrophenyl)-5-phenyl tetrazolium chloride (Alley *et al.*, 1982), was utilized to determine the viability of cells within colonies (>99%).

*Quantitation of cell numbers within colonies grown in agar*

Quantitation of number of cells within clusters or colonies has been delineated for several tumour types grown in soft agar and was measured for each cell type in order to determine minimum colony size from counts based on either visual or automated counts (Meyskens *et al.*, 1984). For CCL 53.1, C8146C, C8161, and R83-4 cell diameters were respectively 13.55, 14.20, 16.44 and 19.40 $\mu$m. The corresponding colony diameter of a 50 cell colony was measured as 77, 82, 97 and 118 $\mu$m, respectively.

*Counting of colonies grown in monolayers*

Petri dishes were stained by adding 1 ml of 0.2% (w:v) methylene blue (AL-DON Chemicals, Rush, NY) to the media. The stain was left on for 30 to 45 min. The media plus dye was aspirated and the plates were carefully washed with deionized water to remove excess dye. Each plate was manually examined and only colonies with 50 or more cells were counted as survivors.

*Radiation*

Cells were irradiated by single dose X-ray generated by a Varian Associates 18 MeV linear accelerator operating at 10 MeV and yielding a dose rate of 5.0 Gray (Gy) min$^{-1}$. A 2.0 cm thick bolus was placed between the source of irradiation and the target. The bolus was placed on top of a single layer of 4 ml snap top plastic tubes containing cells in complete medium. A source to target distance of 100 cm was used, and the cells were irradiated at ambient temperature under normal atmospheric conditions. All radiation dosages and dosimetry readings were provided by the Department of Radiation Oncology of the University Medical Center. Cells were carried to/from the Radiation Oncology Department in an air-tight modular incubator chamber containing 5% $CO_2$ and 95% air.

*Examination for presence of enlarged cells*

In agar individual colonies can be plucked from the Petri dish. Colonies have been removed aseptically by micropipettes pneumatically connected to a microsyringe (Meyskens *et al.*, 1984; Thomson & Meyskens, 1982). Before the colonies were removed 0.5 ml of fresh medium was added to each Petri dish. Each colony was pulled slowly into the micropipette by aspiration of medium ($<5\,\mu$l) with the microsyringe. Individual colonies were stored in the micropipette while other colonies were collected. The colonies were then expelled onto a microscope slide and cell diameters of individual cells were measured. The mean cell diameter was determined by measuring individual cell diameters from at least 10 colonies of each of four size classes >25 cells, >50 cells, >75 cells and >100 cells. The average s.e. was 9%. Mean cell diameter did not vary from one another within the s.e. of control *versus* experimental groups. Enlarged cells were not present in colonies and only observed as single cells at the higher doses. These enlarged cells did not approach the size of the smallest colony diameter (42 $\mu$m) counted by the image analyzer. Therefore, enlarged cells within colonies did not alter the interpretation of our results.

*Model selection, estimation and comparison of survival curves*

Survival data were calculated according to standard radiobiological methods (Elkind & Whitmore, 1967; Puck & Marcus, 1956; Steel & Courtenay, 1983). There were 12 replicates per control and 6 replicates per experimental dose per experiment. $D_0$ values were estimated from the curve fitted by the one-hit multitarget model. Ninety-five per cent confidence intervals were calculated for $D_0$ values. The degree of fit was estimated by the correlation coefficient, $R$ (see **Results** and Table I).

**Table I** Radiobiological parameters and statistical analysis of survival of murine and human melanoma colonies (≥50 cells) grown in bilayer soft agar and in monolayer culture

| | $D_0$ value (Gy)[a] | Agar $D_q$ value (Gy) | n number | R[b] |
|---|---|---|---|---|
| Murine | | | | |
| Cell line CCL 53.1 | 3.19±0.36 | 2.72 | 2.34±0.40 | 0.934 |
| Human | | | | |
| Cell strains | | | | |
| C8146C | 2.15±0.38 | 1.64 | 1.57±0.40 | 0.932 |
| C8161 | 2.16±0.22 | 1.30 | 1.83±0.28 | 0.932 |
| R83-4 | 2.69±0.30 | 2.39 | 2.44±0.47 | 0.927 |

| | $D_0$ value (Gy)[a] | Monolayer $D_q$ value (Gy) | n number | R[b] |
|---|---|---|---|---|
| Murine | | | | |
| Cell line CCL 53.1 | 1.53±0.33 | 0.86 | 1.76±0.53 | 0.929 |
| Human | | | | |
| Cell strains | | | | |
| C8146C | 1.88±0.19 | 0.59 | 1.37±0.18 | 0.914 |
| C8161 | 2.49±0.39 | 3.65 | 3.41±0.97 | 0.948 |
| R83-4 | 2.54±0.42 | 0.52 | 1.22±0.21 | 0.914 |

[a]95% confidence interval and [b]Correlation coefficient.

For each replicated experiment the observed survival data consisted of the mean proportion surviving per dose expressed as a proportion of the control mean. There were three replicated experiments per cell and assay method, with the exception of R83-4 for which only one replicate per assay was possible because no more low passage R83-4 cells were available.

Although several mathematical models have been proposed for analyzing survival data, we chose three 2-parameter models for further analysis. The intent was to fit each of the models to each of the 8 sets of survival data and choose the model which consistently provided the best fit. The following models described in Fertil *et al.* (1980) were examined:

1. $S(D)=1-(1-e^{-D/D_0})^n$ {one-hit multitarget}

2. $S(D)=1-\{1-e^{-D/D_0(1-D/D_0)}\}^n$ {two-hit multitarget}

3. $S(D)=e-^{\alpha D-\beta D2}$ {quadratic}

where $D$ is the experimental dose, and $D_0$, $n$, $\alpha$ and $\beta$ are parameters to be estimated from the data. Estimation was carried out with nonlinear least squares regression (Draper & Smith, 1981) using the SAS statistical package (SAS Institute Inc., 1985).

For each fitted curve the residual sum of squares (RSS) was calculated, which provided a measure of the discrepancy between observed ($S$) and predicted ($\hat{S}$) values as shown in the equation below:

$$RSS=\sum_{i=1}^{k}(S_i-\hat{S}_i)^2$$

where $k$ denotes the number of observations. Since each model has 2 parameters, one criterion for selection was to choose that model with the smallest RSS. Within each cell line and assay method the RSS were ranked from smallest to largest. Friedman's test and Bonferroni multiple comparisons (Conover, 1980) were used to compare the models with respect to RSS. We found that the two-hit model had significantly larger RSS compared with the one-hit and quadratic models. No differences were observed between the one-hit and quadratic models.

The two-hit model was removed from further consideration. Correlation coefficients were calculated between observed and predicted values for the two remaining models. All correlations were at least 0.95, indicating that both models seem to fit quite well. Graphical methods were used to further evaluate the fit of each model. The predicted survival curves and the experimental data were plotted and the residual values ($S-\hat{S}$) were plotted over dose to observe any systematic patterns suggesting lack of fit. These graphical methods indicated both models fit the data reasonably well. We chose the quadratic model for further study because of its biological relevance to radiation survival curves and its ease of use from a data analytic perspective. Table II displays the parameter estimates and estimated standard errors. Figure 1 displays the plots of observed data and predicted survival curves.

**Table II** Parameter estimates and standard errors for the quadratic model by cell line and assay method

| Cell line | Parameter $\alpha(Gy^{-1})$; $\beta(Gy^{-2})$ | Assay method: Agar Est. Std. Err. | Assay method: Monolayer Est. Std. Err. |
|---|---|---|---|
| CCL | $\alpha$ | 0.089±0.025 | 0.105±0.058 |
| | $\beta$ | 0.011±0.004 | 0.110±0.026 |
| C8146C | $\alpha$ | 0.200±0.048 | 0.425±0.033 |
| | $\beta$ | 0.029±0.013 | 0.015±0.012 |
| C8161 | $\alpha$ | 0.178±0.016 | −0.010±0.021 |
| | $\beta$ | 0.029±0.005 | 0.041±0.005 |
| R83-4 | $\alpha$ | 0.067±0.030 | 0.294±0.052 |
| | $\beta$ | 0.027±0.006 | 0.012±0.015 |

Of interest was a comparison of the shapes of the survival curves for the two assay methods for each cell line. We fitted an expanded quadratic model shown below for each cell line.

$$S(D)=e^{-(\alpha_1\alpha_1 G)D-(\beta_0+\beta_1 G)D^2}$$

where $G$ is an indicator variable for assay method, and $\alpha_0$, $\alpha_1$, $\beta_0$, and $\beta_1$ are parameters estimated from the data. A test of the null hypothesis $H_0$ $\alpha_1=\beta_1=0$ provided evidence of the comparability of the shapes of the curves. When $H_0$ was rejected then there was evidence of differences in the curves. It should be pointed out that tests of hypotheses usually carried out with linear models are not directly applicable to the nonlinear case. The above test is only approximate in the nonlinear case. The $P$ values were $<0.001$ for all cells.

Figures 2a–c display the predicted curves and 95% confidence bands for the mean. Figure 2d displays the predicted curves and 95% confidence bands for experimental observations of one experiment. The confidence bands do not take into account the variability among the replicated experiments in Figure 2a–c at a given dose, only the variability associated with estimating the location of the average or expected value. This seems appropriate since we are not interested in prediction for an individual replicate. Caution must be used in interpreting these limits since they are approximations.

### Results

For all cells studied the shape of the survival curve was not the same for cells assayed in the bilayer soft agar or on plastic (Figure 1). All tests of the null hypothesis $H_0$ $\alpha_1=\beta_1=0$ were significant ($P<0.05$) indicating differences in the shapes of the curves. For all cells predicted curves and 95% confidence bands overlap very little between assay methods which suggests differences in the curves (Figure 2). Cells from CCL, C8146C, and R83-4 exhibited a greater apparent sensitivity to radiation in monolayer than in agar (Figure 1a, b and d; Figure 2a, b and d). The reverse was true for cell strain C8161 (Figure 1c and Figure 2c).

**Figure 1** In vitro *radiation survival curves for melanoma colonies* (>*50 cells*). A murine cell line CCL 53.1; B, C, and D, human cell strains, C8146C, C8161, and R83-4. A–C, Mean of 3 experiments are shown; D, Six replicates of one experiment are shown. □. Agar observed values, ——— Agar predicted curve; ○, Monolayer observe values, --- Monolayer predicted curve.

**Figure 2** In vitro *radiation survival curves for melanoma colonies* (>*50 cells*). *Predicted curves and 95% confidence bands*. A, murine cell line CCL 53.1; B, C and D, human cell strains, C8146C, C8161, and R83-4. A–C, confidence bands for the the means of 3 experiments; D, confidence bands for the 6 replicates of one experiment. ——— Agar predicted curve, —— Agar 95% confidence limit; ····· Monolayer predicted curve, ------ Monolayer 95% confidence limit.

For murine melanoma, $D_0$ values were statistically different (see Table I). All human cell strains exhibited $D_0$ values that were not statistically different, although for all cells $D_q$ values and $n$ numbers were different for the two assay systems. In general, $D_q$ values were greater for cells grown in agar than those grown in monolayer. Human cell strain C8161 had higher $D_q$ and $n$ values for the monolayer. Correlation coefficients ranged from 0.914 to 0.934.

### Discussion

For the murine Cloudman S91 cell line CCL 53.1 and human melanoma cell strains C8146C, C8161, and R83-4 the shape of the survival curve was not identical for cells assayed in bilayer soft agar *versus* cells grown on plastic. This would indicate that apparent radiosensitivity is influenced by the assay utilized. Also there is heterogeneity in response of murine and human melanoma cells to radiation, dependent on the assay method.

$D_0$ values were the same (within statistical error) for both monolayer and agar assays for the human melanoma cell strains tested. However $D_0$ values were dissimilar for the murine cell line Cloudman S91 CCL 53.1 as measured in agar or monolayer assay. For murine melanoma cells the $D_0$ value was greater in agar than in monolayer.

In general, $D_q$ values were greater for cells grown in agar than those grown in monolayer. C8161 was the exception to this observation. It is possible that cells grown in agar experienced a greater division delay which allowed cells more time to repair damage before cell division. However, for cells from C8161 the shoulder region was greater for cells grown in monolayer culture than for cells grown in agar, suggesting that for this cell strain cells grown under anchorage-dependent conditions have an advantage in the repair of sublethal damage as well as repair of potentially lethal damage. Melanoma cells which have extensive shoulder regions for radiation survival have been documented by other investigators as well (Rofstad & Bronstad, 1981).

Radiosensitivity is influenced by the cloning assay utilized although this is not apparent in the estimates of $D_0$ values. Differences in apparent radiosensitivity are, however, evident in the estimates of $\alpha$ and $\beta$ values and $D_q$ values. For the CCL, C8146C, and R83-4 strains, cells grown in agar appeared to be more radioresistant than those grown in monolayer.

Comparisons of *in vitro* assay systems have been performed for other cell types. Radiation survival data for human ovarian and cervical carcinoma have been delineated using the Hamburger–Salmon assay. In the paper by West and Sutherland (1986), the soft agar assay of Courtenay–Mills and the Hamburger–Salmon assay were compared. They found for the cervical carcinoma cell line ME180 that the radiation sensitivities were different when assayed by different protocols. This difference was attributed to the existence of sub-populations of resistant slowly growing cells. Furthermore, West and Sutherland theorized that these slowly growing cells were stimulated by additional nutritional factors to produce scorable colonies and that the slowly-growing cells represented the minority. The stimulation of these cells would be masked in the control plates. Therefore, as the radiation dose was increased, the proportion of these resistant cells increased and the $D_0$ of the survival curves increased. Cells appeared to exhibit a greater radiosensitivity in the Courtenay–Mills assay than in the Hamburger–Salmon assay. Likewise, sub-populations of resistant cells may be present in melanoma cells.

Stephens *et al.* (1980) compared the apparent radiosensitivities of anaplastic MT murine cells by monolayer *versus* Courtenay–Mills assay. Cells in the monolayer assay exhibited a greater degree of radiosensitivity although the 95% confidence interval on the $D_0$ and $n$ values overlapped. Stephens *et al.* attributed this apparent difference to the fact that the MT carcinoma had not been adapted for monolayer cloning. The non-adapted tumours, therefore, could contain a proportion of cells which did not survive in monolayer conditions, especially after cytotoxic treatments. The presence of fewer surviving cells in monolayer could be due to this non-adaptation. Our melanoma cells were adapted to agar culture. However, even with this adaptation not all of our cells exhibited a greater degree of radiosensitivity in monolayer. As in the comparison of assay systems for the MT carcinoma cells, there was overlap in the 95% confidence intervals on $D_0$ values for our human melanoma cells even though by comparison of other parameters $D_q$ values and $\alpha$ and $\beta$ parameters, there were obvious differences in the survival curves between assay methods.

Based on the above studies, we would expect that cells assayed in monolayer might exhibit a greater apparent radiosensitivity than in the Hamburger–Salmon assay. In fact, this is the case for the Cloudman S91 murine melanoma and two of the three human melanoma cell strains. These cells may have a portion of the population that grows well in monolayer culture.

In summary, there is heterogeneity in the response of murine and human melanoma cells to single-dose X-ray determined by the measurement assay. $D_0$ values may be the same or dissimilar. In general, $D_q$ values were greater for cells grown in soft agar. Differences in apparent radiosensitivity were influenced by the assay utilized although there was no apparent consistent differences between the curves generated by monolayer or soft agar assay.

The excellent secretarial assistance of Michele Gautreaux and Maggie Pulliam is gratefully acknowledged. We thank Dr E. Gerner for his helpful comments. This work was supported in part by grants from the National Institute of Health (CA 27502, CA 34689, CA 23074) and a Cancer Biology Training Grant (CA 09213-07) to K.Y.

### References

ALLEY, M.C., UHL, C.B. & LIEBER, M.M. (1982). Improved detection of drug cytotoxicity in the soft agar colony formation assay through use of a metabolizable tetrazolium salt. *Life Sci.*, **31**, 3071.

ALPER, T. (1979). *Cellular Radiobiology*. Cambridge University Press: Cambridge.

ASANO, S. & RIGLAR, C. (1981). Colony growth in agar in human melanoma cells. *Cancer Res.*, **41**, 1199.

BARRANCO, S.C., ROMSDAHL, M.M. & HUMPHREY, R.M. (1971). The radiation response of human malignant melanoma cells grown *in vitro*. *Cancer Res.*, **31**, 830.

BREGMAN, M.D. & MEYSKENS, F.L., JR. (1983). *In vitro* modulation of human and murine melanoma growth by prostanoid analogues. *Prostaglandins*, **26**, 449.

BREGMAN, M.D., PETERS, E., SANDER, D. & MEYSKENS, F.L., JR. (1983). Dexamethasone, prostaglandin A, and retinoic acid modulation of murine and human melanoma cells grown in soft agar. *J. Natl Cancer Inst.*, **71**, 927.

BREGMAN, M.D., SANDER, D. & MEYSKENS, F.L., JR. (1982). Prostaglandin $A_1$ and $E_1$ inhibit the plating efficiency and proliferation of murine melanoma cells (Cloudman S91) in soft agar. *Biochem. Biophys. Res. Commun.*, **104**, 1080.

CONOVER, W.J. (1980). *Practical Nonparametric Statistics*. Wiley and Sons: New York.

COURDI, A., GIOANNI, J., LALANNE, C.M. & 4 others (1981). Establishment, characterization, and response to cytotoxic and radiation treatment of three human melanoma cell lines. *In vitro*, **19**, 453.

COURTENAY, V.D., SELBY, P.J., SMITH, I.E., MILLS, J. & PECKHAM, M.J. (1978). Growth of human tumour cell colonies from biopsies using two soft-agar techniques. *Br. J. Cancer*, **38**, 77.

DRAPER, N. & SMITH, H. (1981). *Applied Regression Analysis*. 2nd Ed. Wiley and Sons: New York.

ELKIND, M.M. & WHITMORE, G.F. (1967). *The Radiobiology of Cultured Mammalian Cells*. Gordon and Breach: New York.

ENDRESEN, L., TVEIT, K.M., RUGSTAD, H.E. & PIHL, A. (1985). Chemosensitivity measurements of human tumour cells by soft agar assay are influenced by the culture conditions. *Br. J. Cancer*, **51**, 843.

FERTIL, B., DESCHAVANNE, P.J., LACHET, B. & MALAISE, E.P. (1980). *In vitro* radiosensitivity of six human cell lines. *Radiat. Res.*, **82**, 297.

GOOD, M., LAVIN, M., CHEN, P. & KIDSON, C. (1978). Dependence on cloning method of survival of human melanoma cells after ultraviolet and ionizing radiation. *Cancer Res.*, **38**, 4671.

HAMBURGER, A.W. & SALMON, S.E. (1977). Primary bioassay of human tumor stem cells. *Science*, **197**, 461.

HERMAN, C.J., PELGRIM, O.E., KIRKELS, W.J. & 4 others (1983). In-use evaluation of the Omnicon automated tumor colony counter. *Cytometry*, **3**, 439.

KRESSNER, B.E., MORTON, R.R.A., MARTENS, A.E., SALMON, S.E., VON HOFF, D.D. & SOEHNLEN, B. (1980). Use of the image analysis system to count colonies in stem cell assays of human tumors. *Prog. Clin. Biol. Res.*, **48**, 179.

MEYSKENS, F.L., JR. (1980). Human melanoma colony formation in soft agar. *Prog. Clin. Biol. Res.*, **48**, 85.

MEYSKENS, F.L., JR. (1983). Radiation sensitivity of clonogenic human melanoma cells. *Lancet*, **ii**, 219.

MEYSKENS, F.L., JR., MOON, T.E., DANA, B. & 6 others (1981). Quantitation of drug sensitivity of human metastatic melanoma colony-forming units. *Br. J. Cancer*, **44**, 787.

MEYSKENS, F.L., JR., SOEHNLEN, B.J., SAXE, D.F., CASEY, W.J. & SALMON, S.E. (1981). *In vitro* clonal assay for human metastatic melanoma cells. *Stem Cells*, **1**, 61.

MEYSKENS, F.L., JR, THOMSON, S.P. & MOON, T.E. (1984). Quantitation of the number of cells within tumor colonies in semisolid medium and their growth as oblate spheroids. *Cancer Res.*, **44**, 271.

PAVELIC, Z.P., NOWAK, N.J., SLOCUM, H.K. & RUSTUM, Y.M. (1983). Correlation of tumor-cell growth in four semisolid systems. *J. Cancer Res. Clin. Oncol.*, **105**, 94.

PUCK, T.T. & MARCUS, P.I. (1956). Action of X-rays on mammalian cells. *J. Exp. Med.*, **103**, 653.

ROFSTAD, E.K. & BRUSTAD, T. (1981). Broad-shouldered survival curves of a human melanoma xenograft. *Acta Radiol. Oncol.*, **20**, 261.

ROFSTAD, E.K. & BRUSTAD, T. (1983). Radiosensitivity of the cells of an established human melanoma cell line and the parent melanoma xenograft. *Int. J. Radiat. Biol.*, **44**, 447.

SALMON, S.E., YOUNG, L., LEBOWITZ, J. & 4 others (1984). Evaluation of an automated image analysis system for counting human tumor colonies. *Int. J. Cell Cloning*, **2**, 142.

SAS INSTITUTE INC. (1985). *SAS User's Guide: Statistics*. 5th Ed. SAS Institute Inc: Cary, N.C.

SELBY, P.J. & COURTENAY, V.D. (1982). *In vitro* cellular radiosensitivity of human malignant melanoma. *Int. J. Radiat. Oncol. Biol. Phys.*, **8**, 1235.

SMITH, I.A., COURTENAY, V.D., MILLS, J. & PECKHAM, M.J. (1978). *In vitro* radiation response of cells from four human tumors propagated in immune-suppressed mice. *Cancer Res.*, **38**, 390.

STEEL, G.G. & COURTENAY, V.D. (1983). The radiobiology of human tumour cells. In *The Biological Basis of Radiotherapy*, Steel, G.G., Adams, G.E. & Peckham, M.J. (eds) p. 123. Elsevier Science Publishers: New York.

STEPHENS, T.C., PEACOCK, J.H. & SHELDON, P.W. (1980). Influence of *in vitro* assay conditions on the assessment of radiobiological parameters of the MT tumour. *Br. J. Radiol.*, **53**, 1182.

THOMSON, S.P. & MEYSKENS, F.L., JR. (1982). Method for measurement of self-renewal capacity of clonogenic cells from biopsies of metastatic human malignant melanoma. *Cancer Res.*, **42**, 606.

TVEIT, K.M., ENDRESEN, L., RUGSTAD, H.E., FOSTAD, O. & PIHL, A. (1981). Comparison of two soft-agar methods for assaying chemosensitivity of human tumours *in vitro*: Malignant melanomas. *Br. J. Cancer*, **44**, 539.

VON HOFF, D.D., FORSETH, B., METELMANN, H.R., HARRIS, G., ROWAN, S. & COLTMAN, C.A. (1982). Direct cloning of human malignant melanoma in soft agar culture. *Cancer*, **50**, 696.

WEICHSELBAUM, R.R., NOVE, J. & LITTLE, J.B. (1980). X-ray sensitivity of human cells *in vitro*. *Int. J. Radiat. Oncol. Biol. Phys.*, **6**, 437.

WEININGER, J., GUICHARD, M., JOLY, A.M., MALAISE, E.P. & LACHET, B. (1978). Radiosensitivity and growth parameters *in vitro* of three human melanoma cell strains. *Int. J. Radiat. Biol.*, **34**, 285.

WEST, C.M.L. & SUTHERLAND, R.M. (1986). A radiobiological comparison of human tumor soft-agar clonogenic assays. *Int. J. Cancer*, **37**, 897.